# Hypothalamic Inflammation and Energy Balance Disruptions: Spotlight on Chemokines

**DOI:** 10.3389/fendo.2017.00197

**Published:** 2017-08-14

**Authors:** Ophélia Le Thuc, Katharina Stobbe, Céline Cansell, Jean-Louis Nahon, Nicolas Blondeau, Carole Rovère

**Affiliations:** ^1^CNRS, Institut de Pharmacologie Moléculaire et Cellulaire, Université Côte d’Azur, Valbonne, France; ^2^Helmholtz Diabetes Center (HDC), German Center for Diabetes Research (DZD), Helmholtz Zentrum München, Neuherberg, Germany; ^3^Division of Metabolic Diseases, Technische Universität München, Munich, Germany

**Keywords:** neuroinflammation, hypothalamus, chemokines, energy balance, metabolic diseases, high-fat diet, obesity, anorexia

## Abstract

The hypothalamus is a key brain region in the regulation of energy balance as it controls food intake and both energy storage and expenditure through integration of humoral, neural, and nutrient-related signals and cues. Many years of research have focused on the regulation of energy balance by hypothalamic neurons, but the most recent findings suggest that neurons and glial cells, such as microglia and astrocytes, in the hypothalamus actually orchestrate together several metabolic functions. Because glial cells have been described as mediators of inflammatory processes in the brain, the existence of a causal link between hypothalamic inflammation and the deregulations of feeding behavior, leading to involuntary weight loss or obesity for example, has been suggested. Several inflammatory pathways that could impair the hypothalamic control of energy balance have been studied over the years such as, among others, toll-like receptors and canonical cytokines. Yet, less studied so far, chemokines also represent interesting candidates that could link the aforementioned pathways and the activity of hypothalamic neurons. Indeed, chemokines, in addition to their role in attracting immune cells to the inflamed site, have been suggested to be capable of neuromodulation. Thus, they could disrupt cellular activity together with synthesis and/or secretion of multiple neurotransmitters/mediators involved in the maintenance of energy balance. This review discusses the different inflammatory pathways that have been identified so far in the hypothalamus in the context of feeding behavior and body weight control impairments, with a particular focus on chemokines signaling that opens a new avenue in the understanding of the major role played by inflammation in obesity.

## Introduction

**Figure 1:**
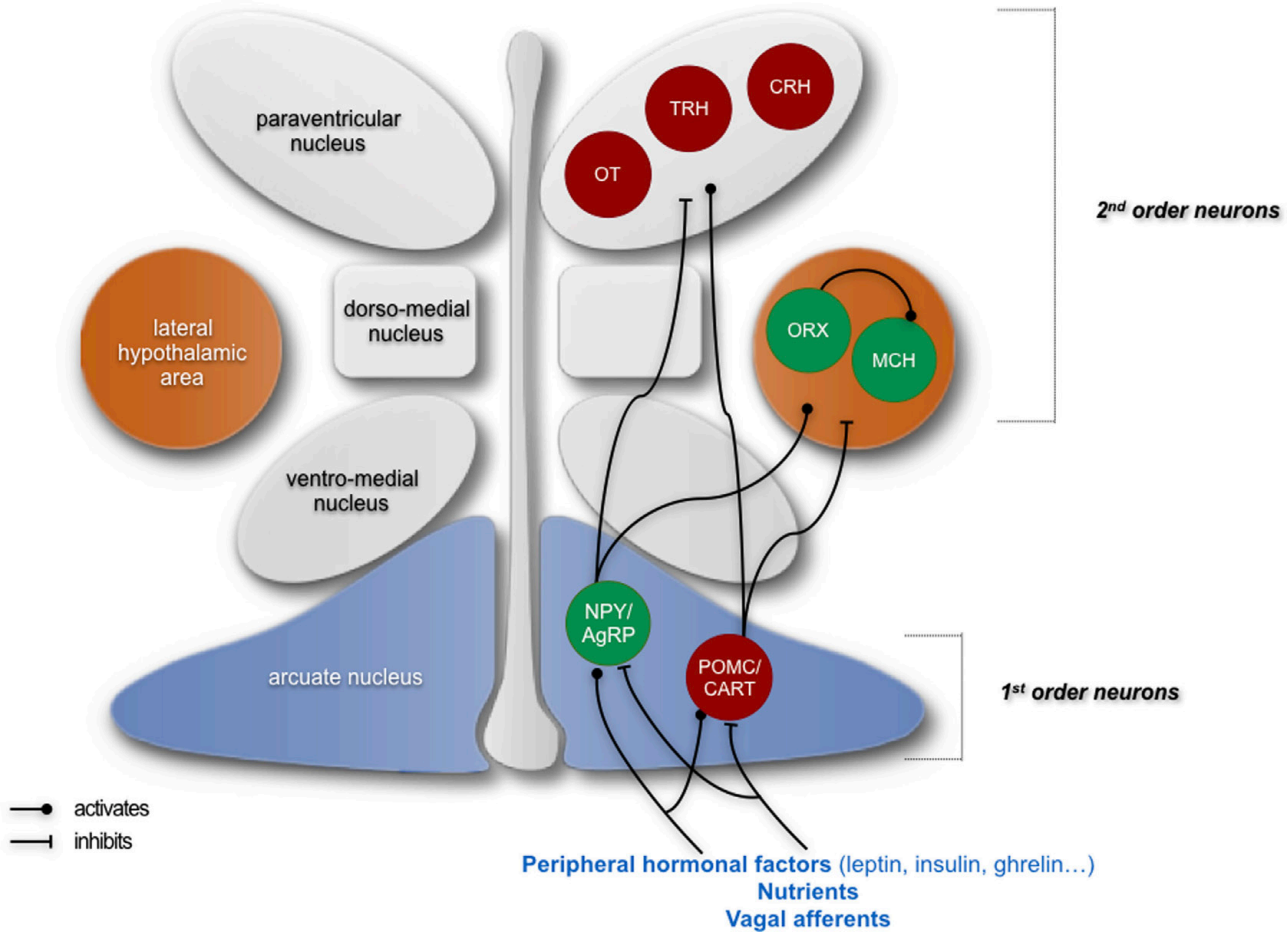
Simplified schematics summarizing the neuropeptidergic hypothalamic networks that are involved in homeostatic regulation of feeding behavior. Signals giving indications about the energy status of an organism are emitted from the periphery and are first integrated by first-order neurons located in the arcuate nucleus, producing either orexigenic neuropeptides (NPY and AgRP) or anorexigenic peptides (POMC and CART). These neurons project to second-order neurons, such as neurons producing the anorexigenic factors oxytocin (OT), thyrotropin-releasing hormone (TRH), and corticotropin-releasing hormone (CRH) in the paraventricular nucleus and neurons producing orexigenic peptides orexin (ORX) and melanin-concentrating hormone (MCH) in the lateral hypothalamus. The integration of peripheral signals by these neuropeptidergic systems participates in the homeostatic regulation of feeding behavior and the maintenance of a suitable weight by ensuring an appropriate food intake as well as appropriate energy intake and expenditure. In green: neurons producing orexigenic peptides; in red: neurons producing anorexigenic peptides. Adapted from Le Thuc and Rovère (7).

Energy balance is finely regulated *via* a bidirectional communication between the brain and the peripheral organs. One brain area is particularly important in this regulation: the hypothalamus. The hypothalamus shelters, in its different nuclei, several neuronal populations producing peptides that are either orexigenic or anorexigenic. The activity of these neuropeptidergic circuits is, among others, modulated by peripheral signals, of neural or hormonal nature, or by nutrients themselves (Figure 1). Thus, it would make sense that the function of these neuropeptidergic circuits would be impaired in case of feeding behavior deregulation, whether it is a loss of appetite or a food overconsumption. Numerous studies, based either on lesion, pharmacological, or genetic approaches, indeed confirmed this [for review see Ref. (1)]. Interestingly, hypothalamic inflammation has already been linked to energy balance disruptions: high-grade hypothalamic inflammation has been associated to involuntary weight loss and, on the contrary, low-grade hypothalamic inflammation has been associated to obesity (2, 3). Importantly, these feeding behavior deregulations represent major public health issues, especially obesity. Indeed, obesity, which keeps developing since the end of the 20th century, is often associated to potentially deadly comorbidities such as diabetes, cardiovascular diseases, liver diseases, and cancers. Yet, a loss of appetite, consecutive to some inflammatory pathologies such as cancer, can also have severe consequences, as it can impair recovery by inducing a deficit in energy.

Hence, understanding the molecular mechanisms linking hypothalamic inflammation and feeding behavior deregulations could, in the long-term, allow identifying potential therapeutic targets. As previously mentioned, we will focus in this review on hypothalamic inflammation, even though peripheral inflammation is also often associated with energy balance deregulations: in involuntary weight loss, inflammation is rather firstly systemic and a consequence of a primary pathology. In obesity, both systemic and hypothalamic inflammations have been described, and even if this is still debated, recent studies suggest that hypothalamic inflammation precedes systemic inflammation associated to the obese state (4–6).

In this context, in particular when there is systemic inflammation, it is important to understand how the periphery and the brain can communicate, especially regarding inflammatory signals. The most described brain–periphery communication pathways are the neural and the humoral pathways. The neural pathway involves primarily the vagus nerve—which expresses cytokine receptors—and the dorso-vagal complex, whereas the humoral pathway involves circulating cytokines that are overexpressed with inflammation and that can (1) bind to their receptors on the membrane of endothelial cells, (2) get to the brain at the level of the circumventricular organs and of the choroid plexus when the blood–brain barrier (BBB) is incomplete, or (3) reach directly the brain thanks to the increased BBB permeability induced by inflammation (8). However, one must also consider the cellular pathway, which involves the infiltration of immune cells and activation of microglia in the brain parenchyma, and also the microbiota pathway, for example.

Studies that aimed to identify inflammatory mediators allowing bidirectional communication between the periphery and the brain during inflammation mainly focused on the role of peripheral cytokines. Yet, it has been suggested that in the context of disruptions of proper control of food intake and body weight, the central production of inflammatory mediators is more important than the peripheral one, or at least “necessary,” in particular in the models of inflammation induced by the bacterial lipopolysaccharide (LPS), where it has been suggested that its effects do not require the signaling from peripheral cytokines (9–11).

Hence, in the context of disruptions of proper energy balance control by hypothalamic circuits, a group of pro-inflammatory mediators might be as relevant as the canonical cytokines, if not more, downstream of the latter: the chemokines. Chemokines are a subgroup of cytokines, small (8–14 kDa) heparin-binding proteins, mainly described for their chemoattractant properties for immune cells to the affected site.

Chemokines and their receptors are suspected to be mediators of the effects of neuroinflammation, from attraction of immune cells to behavioral changes. Beyond participating in the immune response, chemokines and their receptors (the “chemokinergic system”) are constitutively expressed in the brain, in a specific manner, both area wise and cell type wise. Interestingly, it is comparable to the neurotransmitters or neuropeptidergic systems (12, 13). Furthermore, the chemokinergic system has been demonstrated to interact with the neurotransmitter and neuropeptidergic systems (14, 15) and some recent studies suggest that the chemokinergic system is able to directly modulate neuronal activity (16–18). Thus, the chemokinergic system could be responsible for the behavioral changes associated to inflammation.

In this review, we discuss how inflammation, with a focus on chemokines, can participate in the deregulation of the hypothalamic control of energy balance and body weight: first, in appetite and weight loss and, second, in the establishment and/or the development of obesity.

## Involuntary Weight Loss and Inflammation

Weight loss illustrates an unbalance between energy intake and energy expenditure in favor of expenditure. While both involuntary and voluntary weight losses can be caused by a decrease in food intake or an increase in energy expenditure (increase in basal metabolism, e.g., thermogenesis), only an involuntary weight loss can be explained by an uncontrolled decrease in food intake or by an increase in energy expenditure.

There are multiple possible causes for an involuntary weight loss. This weight loss can be a symptom of a severe primary disease such as cancer, where it is particularly preoccupying when it reaches or exceeds 10% in 1 year. In most cases, one can link an involuntary weight loss to psychic troubles, such as depression, that can induce a sustainable loss of appetite, thus weight loss. Excessive consumption of certain substances (e.g., drugs of abuse and alcohol) can also induce an involuntary weight loss. Yet, a weight loss is only considered “involuntary” when it is not a consequence that can be expected from a specific medical treatment of a known medical condition (19). Moreover, aging is often associated to a loss of appetite which induces, in the long-term, severe malnutrition and, obviously, weight loss. This can also be associated to sarcopenia, a geriatric syndrome characterized by loss of muscle mass and function (19). However, as previously mentioned, an involuntary weight loss can have organic causes such as gastrointestinal diseases [e.g., Crohn’s disease, celiac disease (weight loss related to food malabsorption), and digestive ulcer], cardiovascular, endocrine, autoimmune, infectious diseases (e.g., HIV infection, hepatitis, and tuberculosis), neurological diseases (e.g., dementia and Parkinson’s disease), or cancer (19, 20). In the case of cancer, weight loss is one of the first symptoms with 50% of patients with cancer reporting weight loss (21). It should be noted that the majority of the abovementioned organic causes are commonly associated with “high-grade”—in other words high-intensity—inflammation. Interestingly, even if benign and time-restricted, numerous pathological states are strongly inflammatory and associated to a reduced appetite or “anorexia.” This anorexia is part of a classical defense mechanism of an organism against infection, lesions, etc., that is referred to as “sickness behavior” (22, 23). Sickness behavior involves various behavioral changes that primarily affect mood and energy balance, which develop parallel to infection or to another pathology (22).

Like other strategies adopted by the body to promote healing, anorexia is supposed to be temporary and beneficial. However, in the case of chronic diseases such as cancers, both appetite and weight loss can persist, leading to a further degraded state of the subject (hypoglycemic malaise, amenorrhea in women, decalcification leading to more fragile bones, teeth falling, etc.), comparable, in some aspects only linked to undernutrition, to what is observed with anorexia nervosa (24). Prolonged anorexia, irrespective of its type and its causes, is often a contributory factor to the onset of cachexia. Cachexia is a complex syndrome that cannot be fully reversed by conventional nutritional support and leads to progressive functional impairment, i.e., pathological thinness, associated with deep asthenia and organs dysfunction [decreased muscle strength, fatigue, adipose tissue (AT) dystrophy, etc.] (25, 26). Hence, cachexia can be defined as a complex syndrome in which inflammation leads to early satiety and anorexia, decreased fat mass, and weakness (25, 26). Prolonged loss of appetite may even be fatal. One can then assume that inflammation, here high-grade, could disrupt the proper functioning of the hypothalamic systems that are involved, and thus the regulation of feeding behavior. Hence, considering the serious consequences of unresolved anorexia, it is important to better understand the mechanisms linking inflammation and the cerebral centers that control energy homeostasis, because if the link between high-grade inflammation and loss of appetite and weight is established and extensively recognized, the underlying molecular mechanisms have not yet been fully resolved.

### Inflammatory Pathways in the Hypothalamus and Involuntary Weight Loss

In the hypothalamus, some studies have been able to identify some inflammatory pathways, involving different cell types, to be relevant for weight loss.

At the molecular level, as previously mentioned, the literature related to the deregulation of the control of energy balance in the anorexia–cachexia syndrome has focused on the role of pro-inflammatory cytokines, such as interleukin (IL)-1β, IL-6, and TNF-α. It has been shown that pro-inflammatory cytokines, whose production can be induced by infectious agents affecting peripheral organs, notably by LPS—the most commonly used model for modeling disease behavior and anorexia associated with inflammation (27)—participate in the induction of sickness behavior.

Some studies deciphering the kinetic aspects of the effect of central or peripheral cytokine injection suggest that pro-inflammatory mediators exert their effect primarily at the central level versus periphery (28, 29). Numerous studies have focused on IL-1β and have shown that its intracerebroventricular (ICV) injection induces profound behavioral changes in rodents (30). Furthermore, the central role of cytokine signaling at the central level has been confirmed, for example, by the fact that the central injection of the IL-1β receptor antagonist prevents the effects induced by the peripheral injection of this cytokine (31, 32). In addition, some studies suggest that inflammatory inducers such as LPS are not capable of directly inducing sickness behavior and require downstream actors such as cytokines. Indeed, for example, unlike the central injection of LPS, central injection of IL-1 is able to induce sickness behavior in mice whose LPS toll-like receptor (TLR) 4 is non-functional (33).

As mentioned earlier, cytokines, involved in the induction and regulation of sickness behavior at the central level, can be released into the circulation by the immune cells (8) or directly produced by neurons and glial cells in the central nervous system (CNS) (34–39). In the context of systemic diseases, the central effects of these cytokines appear to be independent of the place of their secretion. As previously mentioned, when produced in the periphery, cytokines have the following two main pathways to act on the brain: a neural pathway and a humoral pathway. Most cytokines act in a paracrine manner at the site of infection, suggesting that neural afferents may be the target of pro-inflammatory cytokines. Interestingly, the perineural sheath of the vagus nerve contains immune cells that are capable of producing IL-1, in particular in response to LPS (40). Furthermore, the sensory neurons of the vagus nerve express IL-1 receptors and it has been shown that this cytokine stimulates the sensory activity of the vagus nerve (40). Vagotomy experiments have demonstrated the importance of the vagus nerve in the transmission of information from the periphery to the brain: for example, after injection of LPS or IL-1 at the periphery, vagal afferents are involved in the induction of the sickness behavior and in the neural activation of the brainstem, the hypothalamus, and the limbic structures (40).

Chronic administration of cytokines can reproduce the characteristics of anorexia–cachexia syndrome (38, 41–44), while blocking the signaling of one of them, such as TNF, by the use of neutralizing antibodies, inhibits its development (42, 45, 46). Similarly, administration of an IL-1 receptor antagonist prevents anorexia in cancer animal models (47). Some studies have suggested that endogenous brain IL-1 is a mediator of LPS-induced anorexia by acting on the expression of cytokines in the hypothalamus (9). In addition, it appears that IL-1β can act on ARC POMC neurons (48) and that TNF-α indirectly increases energy expenditure *via* β3 adrenergic signaling in the brown AT (thermogenesis) (49). By different approaches, it has been shown that interfering with mediators of inflammation coincides with a reduction in hypothalamic inflammation and prevents weight loss in animal models of anorexia: for example, the inhibition of the “adenosine monophosphate protein kinase” in the hypothalamus reduces hypothalamic inflammation, which is accompanied by an increase in food intake in the case of cancer-associated anorexia, allowing better overall survival (50). Interestingly, in the context of cancer-associated cachexia, the administration of ghrelin, an appetite-stimulating hormone, increases food intake and is accompanied by a decrease in IL-1β (51).

Focusing on the LPS-induced anorexia model, it is interesting to underline that some studies suggest that this loss of appetite is independent from the vagal afferents and that it only depends on central inflammatory mechanisms, where the central effects of peripheral LPS could be, among others, mediated by some cytokines and/or *via* receptors for LPS expressed by some brain cells (11, 52). Indeed, microglia expresses TLR4, through which LPS has been shown to exert some of its effects (53–56). Furthermore, Hines and colleagues have shown that disrupting TLR4 signaling prevents microglial activation, inhibits the production of cytokines, and prevents the development of the sickness behavior that are induced by peripheral LPS (57). In contrast, a recent study by Reis et al. has shown that if microglia and TLR4 are necessary for LPS to acutely induce inhibitory effects on orexigenic agouti-related peptide (AgRP)/neuropeptide Y (NPY) neurons, conversely, LPS can actually acutely increase the firing activity of anorexigenic pro-opiomelanocortin (POMC) neurons in a microglia/TLR4-independent manner (58). In addition, the same study presents results which further supports that mediators of inflammation such as microglia and TLR4 can, at least acutely, affect basal food intake and hormonal-dependent modulation of feeding behavior. Indeed, the inhibition of microglia alone, *via* ICV injection of minocycline, leads to an increase in food intake, comparable to the one induced by ICV ghrelin (58). Interestingly, the co-injection of ghrelin and minocycline does not synergistically increase food intake. This implies that the inhibition of microglia itself, as it increases food intake, interferes or prevents ghrelin’s orexigenic effects (58).

Changes in gene expression of hypothalamic anorexigenic and orexigenic neuropeptides were assessed in mice or rats that received LPS IP injections (59–61). Generally, peripheral LPS appears, initially, to reduce the expression of the orexigenic peptides and, on the contrary, to increase that of the anorexigenic peptides. In later stages, the expression of orexigenic peptides increases again, probably to promote food intake and compensate for weight loss.

Some authors have used other models to study to understand links between inflammation in the brain and the induction of sickness behavior.

For example, Jang and colleagues sought to determine the mechanisms behind anorexia and weight loss associated with illness *via* administration, in mice, of either bacterial or viral products: LPS and human immunodeficiency virus-1 transactivator protein (Tat), respectively (62). They found in both cases that pro-inflammatory cytokines such as IL-β, IL-6, and TNF-α were upregulated in the hypothalamus. Furthermore, AtT-20 and SH-SY5Y cells treated with either Tat or LPS exhibit increased POMC transcriptional activity. In addition, the injection of Tat or LPS in the hypothalamus of mice induces in both cases a decrease in food intake and in body weight. They identified POMC as a potential mediator of illness-induced anorexia and as a possible downstream target of NF-κB. Indeed, they showed through different approaches that the NF-κB pathway in the melanocortin system plays an important role in illness-induced anorexia and body weight loss: both administration of AgRP, an endogenous melanocortin antagonist and the inhibition of the NF-κB pathway specifically in the POMC neurons (Ikkβ^ΔPomc^ mice), significantly blunted the effects of Tat and LPS on food intake and body weight. Interestingly, the authors also present results suggesting that leptin-induced anorexia is also dependent on the NF-κB pathway (62).

Another study has shown that a pathway involving TLR2 participates in the induction of sickness behavior through a microglia-POMC neurons axis (63). In this publication, the authors show, by ICV injection of Pam3CSK4, a specific synthetic TLR2 agonist that TLR2 central signaling is able to trigger sickness behavior through activation of microglial cells, which express TLR2, and *via* the NF-κB and COX pathways. The authors also show that activation of central TLR2 is able to modify the synaptic architecture in the ARC, especially at the level of the POMC neurons: it reduces the rate of GABAergic contacts on POMC neurons, whereas it increases the vesicular glutamate transporter 2 (vGLUT2) contacts on POMC soma, translating an increased excitatory state, in correlation with an increased microglial occupancy.

In addition, Murray et al. used the peripheral administration of the viral mimetic poly I:C to induce type-I interferons (IFN-I) overexpression in the brain. Using IFN-I receptor 1 (IFNAR1)-deficient mice, the results of the authors present suggest that IFN-I are involved in the induction of sickness behavior, including anorexia, whereas IL-6 participates in sickness behavior but not in anorexia (64).

Furthermore, a local increase in serotonin levels in the hypothalamus has already been linked to anorexia and cachexia (65–67) and a recent study showed that inflammation induced by peripheral treatment by either IL-6 and/or TNF-α is associated with an impairment in local serotonin turnover in the hypothalamus, a decrease in NPY and AgRP gene expressions, and a decrease in food intake in comparison to control conditions (68). Authors were able to identify upstream inflammatory regulators including interferon gamma (IFN-γ), transforming growth factor beta (TGF-β), IL-6, and IKBKG, an enzyme crucial for the activation of the NF-κB pathway. This study thus suggests that peripheral inflammation reaches the hypothalamus where it impairs serotonin turnover, which is associated to a decrease in food intake.

Taken together, numerous studies support the hypothesis that inflammation at the hypothalamic level is able to disrupt the proper function of neuropeptidergic circuits of the hypothalamus and thus to induce an involuntary weight loss.

However, mediators linking inflammation and its consequences at the level of central systems regulating energy homeostasis, and ultimately on weight, have not been determined with certainty, and one may note that the nature of the mediators responsible for the central effects of IL-1β has been especially poorly described. Thus, chemokines can represent interesting candidates to study further understanding of the underlying mechanisms.

#### Chemokines in the Modulation of Hypothalamic Neuropeptidergic Circuits in Inflammation-Associated Involuntary Weight Loss

So far, very few studies have sought to determine if chemokines have a role in the induction of involuntary weight loss associated with inflammation by impairing the homeostatic regulation of energy balance by hypothalamic neuropeptidergic circuits.

A study published in 1994 by Plata-Salamán and Borkoski aimed to investigate how chemokines could act on the regulation of feeding. Indeed, as previously mentioned, chemokines are produced in multiple types of cells as a response to pathological conditions such as infection, inflammation, injury, and trauma. Among the stimuli that can induce the release of these chemokines, we can list LPS, and also the IL-1β, TNF-α, and IFN, which have been associated to food intake suppression by direct action on the CNS. Thus, the authors aimed to determine if chemokines could be involved, as downstream mediators, in the decrease in food intake induced by inflammatory signals such as LPS. In order to do so, they used, in rats, ICV microinfusion of different chemokines from two different subfamilies: CXC and CC, also known as the α and β subfamilies. This way, they tested the effect of CXC-motif chemokine ligand (CXCL)-1, 4, 7, 8, 10 and CC-motif chemokine ligand (CCL)-2–4, 5. Even though it is at a lesser extent than the cytokine IL-1β, their results indeed identify certain chemokines as capable of acutely decreasing food intake: CXCL4, CXCL8, CXCL10, CCL2, and CCL5. Interestingly, in their model, these chemokines would affect feeding behavior at different time scales: if they all reduced food intake in the 2 h following injection, only CXCL8, CXCL4, and CCL2 were able to reduce food intake over the whole dark phase and only CXCL8 and CXCL4 were able to decrease the total daily food intake (69). This study is particularly important as it sets chemokines as contributors, in the brain, to the effects of infection/inflammation on feeding behavior. Nevertheless, no mechanistic insights were then provided.

As previously acknowledged, very few studies aimed at better characterizing how chemokines could modulate feeding behavior after this study by Plata-Salamán and Borkoski.

Yet, in a recent study from our laboratory (70), we sought to identify and characterize chemokines that could possibly deregulate the hypothalamic circuits to alter food intake and energy balance in anorexia and weight loss. We thus assessed the gene expression of several pro-inflammatory mediators in the hypothalamus of mice that had received an acute ICV injection of LPS. After confirmation of LPS-induced inflammation in our mice by observing the overexpression of several pro-inflammatory mediators, we then identified ligands of the chemokine receptors CCR (CC-motif chemokine receptor)-2 and 5 as the most overexpressed. Among them, CCL2 (also known as monocyte chemoattractant protein 1) caught our attention as it has been described as particularly important in the context of LPS-induced neuroinflammation and suggested to be able to reduce food intake (69, 71–73). Thus, we focused on the central signaling of CCL2 and its receptor CCR2 and could demonstrate that it is mandatory for both metabolic and behavioral changes induced by LPS (70). Indeed, inhibiting CCR2 signaling by combined ICV injection of a specific antagonist of CCR2, INCB3344, together with LPS, prevents the weight loss that is induced by ICV injection of LPS alone. Similarly, the weight loss induced by ICV injection of LPS is reduced in mice deficient for CCR2 in comparison to control animals. Experiments in metabolic cages demonstrate that central injection of LPS decreases both food intake and locomotor activity, whereas it increases fat oxidation and induces a shift in used energy substrate in favor of lipids versus carbohydrates. These two last points probably illustrate an increased use of the energy stocked in the AT as lipids. Interestingly, these LPS effects highlighted by the metabolic cages experiments were also reduced when interfering with central CCR2 signaling by co-ICV injection of LPS and INCB3344.

We then identified the neurons, which produce melanin-concentrating hormone (MCH), a peptide known to elicit food intake and to decrease energy consumption, as targets for CCL2 (74). After ICV injection of LPS, we observed a sustained decrease in gene expression and protein levels of MCH, which is blunted/prevented by pharmacological (using INCB3344) or genetic inhibition of CCL2 signaling. Interestingly, we found that, similar to LPS, ICV injection of CCL2 promotes neuroinflammation, together with a decrease in both MCH expression and body weight. Immunostaining experiments showed that 70% of the MCH neurons of the lateral hypothalamus (LHA) express CCL2 receptor. These neurons responded to CCL2 by decreasing both electrical activity and MCH release. Thus, it seems that the inhibition of the MCH system by LPS depends primarily of the central signaling of CCR2. Moreover, in experiments of perifusion of hypothalamic explants treated with KCl and CCL2, MCH secretion is totally inhibited, while co-application of KCl and INCB3344 only, leads to enhanced secretion of MCH. This suggests that endogenous CCL2 could participate, in normal conditions, in modulating MCH system and thus energy balance regulation.

**Figure 2:**
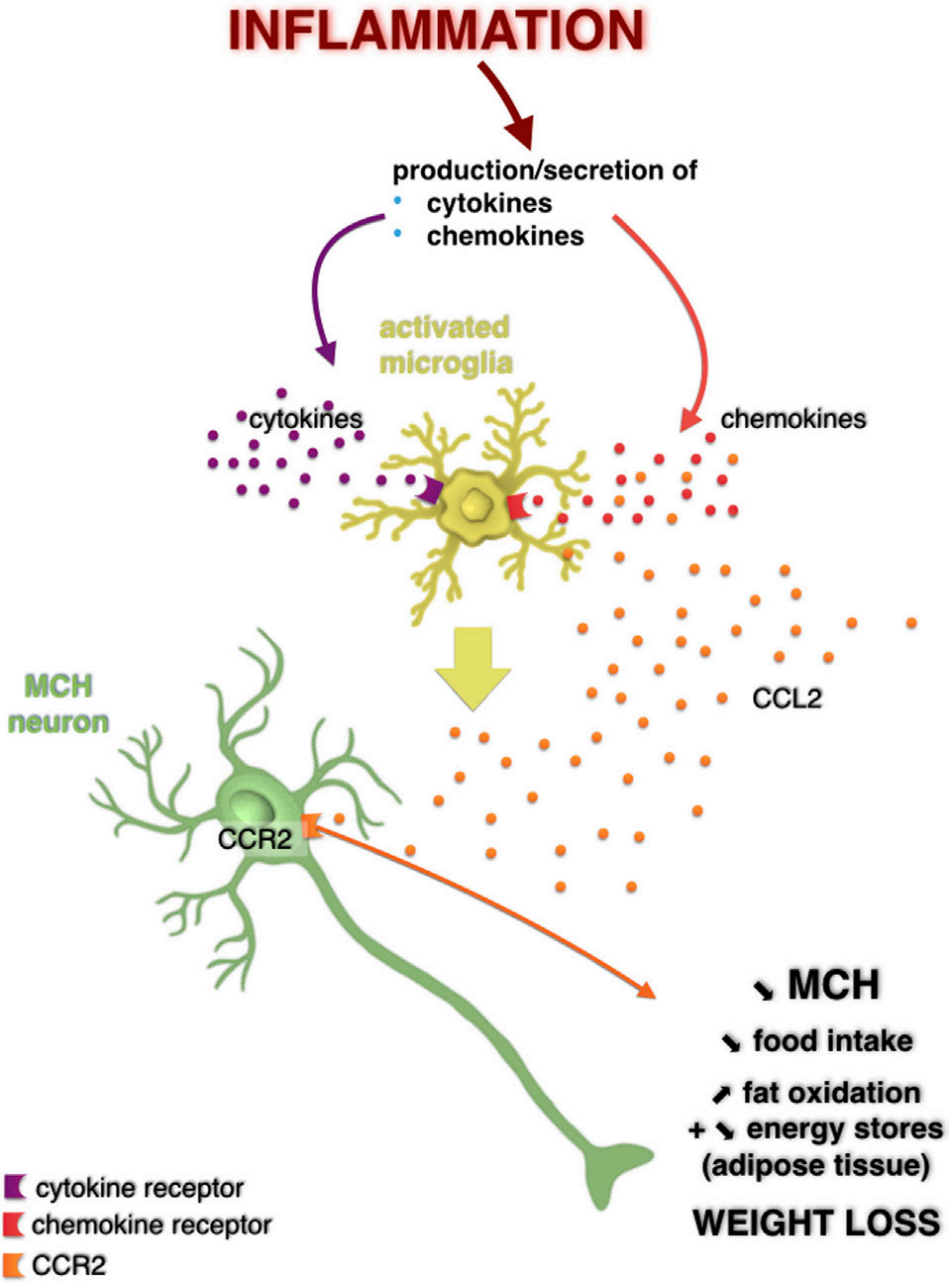
Potential action of the CC-motif chemokine ligand (CCL) 2/CCR2 signaling pathway on melanin-concentrating hormone (MCH) neurons in a weight loss model induced by a central injection of lipopolysaccharide. Hypothalamic inflammation is characterized by overexpression of inflammatory mediators such as cytokines and chemokines. It is possible that these bind to their receptors expressed by glial cells such as microglia, which then activated, can produce even more cytokines and chemokines, including CCL2. However, it is not excluded that CCL2 could act directly on MCH neurons that expressed its receptor in the lateral hypothalamus. This would result in a decreased MCH neuronal activity and in a decreased secretion of this neuropeptide, which is associated to a loss of appetite, an increased fat oxidation, likely reflecting a decrease in energy stores in adipose tissue, and thus a loss of weight. Adapted from Le Thuc and Rovère (7).

**Figure 3:**
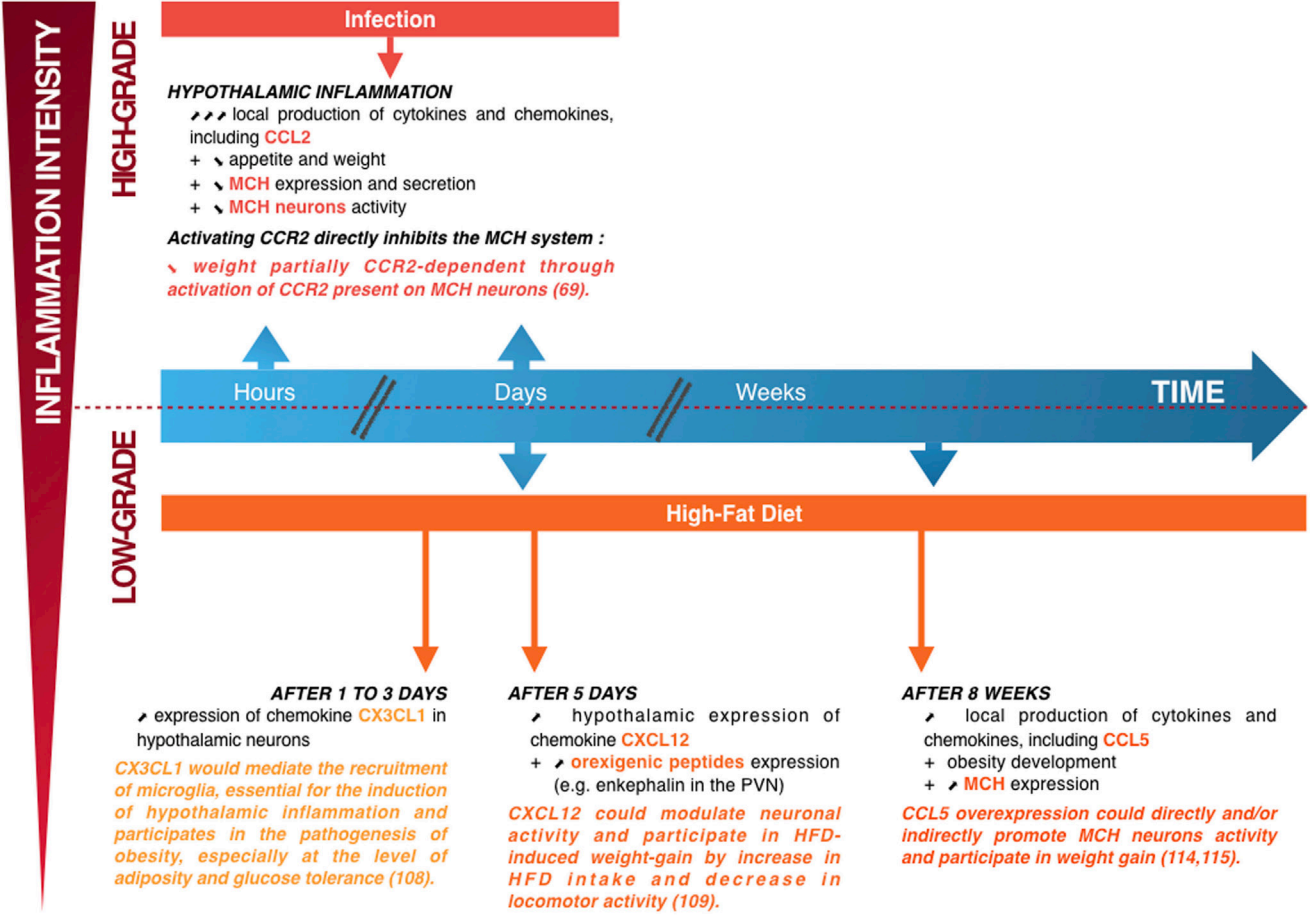
Summary diagram showing the links between hypothalamic inflammation and deregulation of weight. *Upper part*: when lipopolysaccharide induces high-grade hypothalamic inflammation, there is an acute and increase in local production of cytokines and chemokines, including CC-motif chemokine ligand (CCL) 2 (a few hours to a few days) associated with weight loss (a manifestation of the sickness behavior associated with a high-intensity infection). This weight loss is partially dependent on the CCL2/CCR2 signaling pathway, by direct inhibition of the lateral hypothalamus (LHA) neurons producing the orexigenic peptide melanin-concentrating hormone (MCH) (70). *Lower part*: short (1–5 days) or prolonged (several weeks) high-fat diet (HFD) consumption is associated with chronic low-grade hypothalamic inflammation that appears to be linked to the development of obesity, together with an overexpression of orexigenic neuropeptides such as enkephalin in the paraventricular nucleus or MCH in the LHA. The chemokine CX3CL1 appears essential to the induction of hypothalamic inflammation, with an important role in the recruitment of microglia, whereas the chemokines CXC-motif chemokine ligand 12 and CCL5 could modulate neuronal activity and participate in HFD-induced weight loss (75–78). Adapted from Le Thuc and Rovère (7).

Thus, it appears that the central CCL2/CCR2 axis is able to directly act on neurons producing the orexigenic peptide MCH by reducing their activity and both the expression and secretion of the peptide, leading to a reduced food intake and to an increased use of energy stores. Hence, the central CCL2/CCR2 axis, by acting through MCH neurons, appears as a major actor in appetite and weight loss associated with LPS-induced inflammation (Figures 2 and 3). This study was the first to demonstrate that a chemokine can play a role, at the central level, in energy balance deregulation, by acting directly on neuropeptidergic systems in the hypothalamus.

## Obesity and Inflammation

As previously mentioned, interestingly, inflammation is also a characteristic for an opposite disorder of the energy balance disorder of the regulation of food intake and body weight: obesity. Yet, different from the inflammation associated to involuntary weight loss, this inflammation is of low intensity, also referred to as a “low-grade” inflammation (79). Obesity is characterized by an excessive fat mass distributed throughout the body that can be harmful to health [source: World Health Organization (WHO)]. One is considered obese if one’s body mass index is greater than or equal to 30 (80, 81). Worldwide, close to 13% of the population were obese in 2014 and the prevalence of obesity nearly doubled between 1980 and 2014 (source: WHO). Obesity is a concern as it represents a risk factor for chronic diseases such as cardiovascular diseases (hypertension, heart disease, and stroke), diabetes, hepatic steatosis, respiratory diseases, musculoskeletal disorders (osteoarthritis, etc.), certain cancers (endometrium, breast, colorectal, etc.), and neurodegenerative disorders (79–81). Furthermore, childhood obesity, which is progressing in an alarming manner, can promote respiratory difficulties, high blood pressure, the emergence of markers of cardiovascular disease, fractures, insulin resistance, and psychological problems. All these aspects in turn increase the risk of adult obesity, premature death, and disability in adulthood (source: WHO).

While the development of obesity may be explained by some genetic aspects or be consecutive to a primary disease and/or its treatment (hormonal and/or psychological factors, drugs, etc.), its most common cause is a change in the population’s life-style. This encompasses an increase in sedentariness and hypercaloric diets overconsumption, where the excess in calories intake most often comes from lipids and also carbohydrates (80, 81). As obesity represents a major and growing public health issue, it becomes important to identify and understand its causes and mechanisms. Therefore, understanding the role played by inflammation, both peripheral and central, in the establishment and/or development of obesity and its comorbidities might allow to identify new potential targets in the fight against obesity.

The inflammation associated to obesity exhibits several specificities: first, as mentioned previously, it is low grade and chronic. So far, it has been mostly described in peripheral tissues (AT, liver, pancreas, etc.). Nevertheless, more recently, it has been shown to also occur in the CNS: a hypercaloric challenge, especially a high-fat diet (HFD), even on the short term, can induce an inflammation in the hypothalamus that is sustained in models of nutritional obesity (82).

### Obesity and Peripheral Inflammation

Studies have associated inflammation and obesity for a long time (79). Yet this inflammation is not “typical,” as it cannot be associated to the cardinal signs of redness, swelling, heat, and pain. Thus, the inflammation associated to obesity is of different nature. First, it is aseptic: it is caused by the overconsumption of specific nutrients (lipids and carbohydrates); thus, the trigger of this inflammation can be considered to be of metabolic nature. Interestingly, not only the trigger of this inflammation is metabolic, but this inflammation first targets the cells that specialized in metabolism: e.g., adipocytes, hepatocytes, pancreatic β-cells, and myocytes (but also the neurons of the peptidergic systems involved in the regulation of feeding behavior, as detailed further). Hence, this inflammation is often referred to as “metabolic inflammation” or “metainflammation” (79).

The characteristics of the inflammation associated with obesity, in periphery at least, are: (i) being of metabolic nature—it is induced by nutrients and is orchestrated by “metabolic” cells; (ii) being low grade, with moderate and localized overexpression of pro-inflammatory mediators; (iii) creating an impaired milieu where the tissue “composition” in terms of immune cells favors an inflammatory environment in the tissues; and (iv) being sustained in time, without any apparent resolution (79). Furthermore, peripheral metabolic inflammation associated with obesity is deleterious at many levels and, as a consequence of poor eating habits favoring the obese phenotype, inflammation itself promotes tissue dysfunctions, which also contribute to development of obesity, including resistance to insulin and leptin.

### Obesity and Hypothalamic Inflammation

In the context of nutritional obesity, inflammatory pathways are not only activated in peripheral tissues but also activated in central areas involved in the control of energy metabolism, especially in the hypothalamus. Several questions then deserve to be asked and answered such as “What are the signals that induce this inflammation?” and “Is inflammation a cause or a consequence of obesity?”

The relationship between nutritional obesity and hypothalamic inflammation was first described by De Souza and his collaborators (83). The authors demonstrated in rats that a 4-month period of HFD feeding activates inflammatory pathways in the medio-basal hypothalamus (MBH), such as JNK and NF-κB, leading to the production of canonical pro-inflammatory cytokines such as IL-1β, TNF-α, and IL-6 and to deficiencies in insulin and leptin signaling. These observations on hypothalamic inflammation associated to nutritional obesity were confirmed in rats, obese mice, and non-human primates (82, 84–92). Interestingly, some studies have shown that the inflammation induced by the consumption of HFD, likely a response to overnutrition, develops much faster in the hypothalamus than in the peripheral tissues. Indeed, inflammation in the AT, for example, is only observable after several weeks or even months, whereas in the hypothalamus, the consumption of a HFD during a much more restricted period of 24–72 h is enough to induce overexpression of pro-inflammatory cytokines and gliosis—which corresponds to an activation and proliferation of glial cells such as microglia and astrocytes, an inflammation hallmark in the brain (82).

Thus, it appears that the inflammation induced by the excess of nutrients in the hypothalamus precedes the establishment of obesity, as well as peripheral inflammation and metabolic disturbances. It is worth mentioning that the gliosis induced by the consumption of a HFD for a short-period of time (2–3 weeks) is actually reversible, whereas it reappears after longer periods of HFD consumption (8 months) (82). This suggests that the initial short-term gliosis is rather a protective mechanism induced to protect against the “injury” induced by the overload of fat, which is in line with the known roles of these cells, and that with time, the consumption of HFD triggers a durable gliosis and inflammation that would then play a major role in the development of the pathophysiology induced by overnutrition. For example, a study by Dalvi et al. further supports that acute gliosis associated with a moderate period of HFD feeding would actually be protective: as long as the inflammation is restricted to glial cells, the organism seems to attempt to limit brain injury by overexpressing in parallel some protective factors such as the heat shock protein 70 and the ciliary neurotrophic factor. After a longer period of HFD feeding, overexpression of inflammatory mediators within the neurons suggests an exhaustion of neuroprotective mechanism in the hypothalamus, leading to a deregulation of the expression of certain neuropeptides that will favor a positive energy balance (93).

What is the signal that triggers hypothalamic inflammation? It has been suggested that it is the excess of nutrients, which are major physiological regulators of hypothalamic neural networks, that induces the establishment of inflammation at the level of the hypothalamus. Some studies attribute the triggering of hypothalamic inflammation to, more especially, saturated fatty acids (SFA), especially long-chain SFA, due to their accumulation in the hypothalamus when consuming HFD, which could, among others, induce inflammatory signaling by activating the TLR4 pathway. It is interesting to note that other fatty acids, such as long-chain polyunsaturated fatty acids, appear, conversely to long-chain SFA, to be beneficial, especially in the diet-induced obesity (DIO) context, as they have anti-inflammatory properties (94–97). In contrast, not only nutrients but also hormones seem to play a role in the regulation of glial cells function. Indeed, as illustrated by a study by Gao et al., showing that monogenic obese mice with deficient leptin signaling [*ob/ob* (leptin deficient) and *db/db* (leptin receptor mutation)] exhibit less microglial activation than wild-type controls both on chow and on HFD (98). The authors further demonstrated that the lack of leptin signaling affects also the microglial function in the hypothalamus as the expression of several inflammatory mediators is reduced (98). This indicates that the signaling of leptin, already described as a pro-inflammatory adipokine (99), could affect microglial activation. Other mechanisms have been proposed as mediators of the hypothalamic inflammation associated with overnutrition such as the endoplasmic reticulum stress (86, 100, 101). Similarly, oxidative stress could be an initiating factor and participate in the maintenance of the hypothalamic inflammation induced by nutrition. Indeed, at the periphery, oxidative stress, along with the production of reactive oxygen species (ROS), has been shown to precede severe metabolic disturbances and insulin resistance (102). Yet, the brain alone uses a large amount of the oxygen and calories consumed by the body, making it particularly vulnerable to excessive ROS production and oxidative stress. Indeed, mitochondria naturally produce ROS in physiological conditions, especially during the oxidation of nutrients such as glucose or fatty acids (103), and it has been shown that an excessive production of ROS in the hypothalamus occurs in the obese rat. This impairs not only the local detection of glucose but also the associated physiological response, such as peripheral insulin secretion (104). Autophagy is another possible pathway. It is a cellular process allowing the elimination of damaged cytoplasmic elements and organelles in order to maintain internal homeostasis and structural integrity and also plays a key role in cellular responses to metabolic stress. Under conditions of overnutrition, endoplasmic reticulum stress and oxidative stress can induce autophagy (105, 106). Impairing the pathways of autophagy can increase food intake and weight, in association with overactivation of the IKKβ/NF-κB pathway in the hypothalamus, illustrating the connection between autophagic and inflammatory pathways in the hypothalamus.

Over the recent years, the importance of the role played in hypothalamic inflammation in the onset of metabolic disorders in obesity was indeed underlined and the potential of counteracting inflammation in the hypothalamus as a strategy in the fight against obesity was highlighted.

For example, a recent study by Douglass and colleagues demonstrates that astrocytes in the MBH of HFD-fed mice mediate hypothalamic inflammation together with DIO *via* their own inflammatory signaling (107). Indeed, the authors showed that the inducible and specific deletion of IKKβ in astrocytes, which should blunt astrocytic inflammatory capacity, reduces the mice susceptibility to DIO (reduced weight gain and fat mass associated with a decrease in food intake and an increase in energy expenditure), improves glucose tolerance and insulin sensitivity (according to glucose and insulin tolerance tests), and finally, reduces HFD-induced astrocytosis in the MBH.

Furthermore, it has been shown that subcutaneous application of liraglutide or canagliflozin (an inhibitor of the sodium-glucose cotransporter 2) in obese and insulin-resistant rodents is able to disrupt the activation of microglial cells in the hypothalamus, which is associated with an improvement of insulin and glucose homeostasis. In addition, when the ICV injection of IL-4 in HFD-fed rats increases further HFD-induced inflammation in the hypothalamus and causes excessive weight gain, the ICV injection of an IKKβ/NF-κB blocker allows, on the contrary, to prevent hypothalamic inflammation, which is associated with both a decrease in body weight and fat mass and improvements in glucose metabolism and general energy homeostasis in DIO animals (86, 108–111).

Another study by André et al. shows that overnutrition will induce, in addition to an increase in body weight and in adiposity, an increase in the number of microglial cells in the ARC. The authors show the inhibition of microglia expansion in the ARC, achieved by the central delivery of an antimitotic drug, allows to limit food intake and the increase in body weight and in adiposity and also to restore leptin sensitivity. This is associated with a “predictable” inhibition of the upregulation of inflammatory pathways in the ARC that is normally associated with overnutrition, but also in periphery (112).

Taken together, studies studying the hypothalamic inflammation associated with obesity support that this inflammation plays a key role in the metabolic dysfunctions associated with obesity and represents a very interesting target.

Research aiming to understand how the central regulation of energy balance is altered in the context of overnutrition has mainly focused on neurons so far. Yet, as previously mentioned, since hypothalamic inflammation, especially its gliosis feature, has been associated to overnutrition, a strong interest in the role of glial cells (mainly microglia and astrocytes) emerged more recently. Interestingly, these glial cells, once activated, are capable of overproducing, locally, pro-inflammatory mediators such as cytokines and chemokines that can, in turn, affect the neuropeptidergic systems of the hypothalamus, potentially participating in the onset of obesity.

Interestingly, chemokines and their receptors have been demonstrated to be expressed by glial cells, and also by neurons. Especially, chemokine receptors appear to be more expressed by neurons than cytokine receptors in some nuclei of the hypothalamus, allowing to ask the following question: “Could not chemokines act as one of the latest inflammatory mediator that would link inflammation to the disruption of the proper functioning of the neuropeptidergic systems involved in the regulation of the energy balance to promote weight gain and thus the development of obesity?”

#### Chemokines in the Modulation of Hypothalamic Neuropeptidergic Circuits in DIO

As it was the case in the context of hypothalamic inflammation and involuntary weight loss, only few studies have aimed to determine and understand if and how chemokines are able to alter the function of hypothalamic neuropeptidergic circuits and thus participate in the development and/or the maintenance of obesity.

A study by Morari and colleagues has identified fractalkine (or CX3CL1) as involved in the early activation of hypothalamic inflammation in a murine model of DIO, likely through recruitment of microglial cells which express CX3CR1, the receptor for CX3CL1 (75). In this study, the authors show that, early after HFD introduction to the mice (1–3 days), CX3CL1 is induced in hypothalamic neurons of obesity-prone mice, unlike what was observed in obesity-resistant mice. Interestingly, the inhibition of CX3CL1 in the MBH by an approach of small interfering RNA allows a reduction in inflammation, glucose intolerance, and diet-induced adiposity (no significant difference in body weight was observed though). It suggests that CX3CL1, mediating early recruitment of microglia induced by HFD and thus participating in the induction of hypothalamic inflammatory response, participates in the pathogenesis of obesity as it impairs glucose tolerance and adiposity (Figure 3).

In addition, one recent study highlights the role of the axis CXCL12/CXC-motif chemokine receptor 4 (CXCR4) in the paraventricular nucleus (PVN) of the hypothalamus in mediating both neuronal and behavioral effects of the consumption of a HFD in rats (76). In more details, the authors show that a 5-day consumption of a HFD induces an overexpression of CXCL12 and its receptors CXCR4 and CXCR7 both in the PVN and in the perifornical lateral hypothalamus (PFLH). The authors also show that HFD is able to induce an overexpression of orexigenic neuropeptides enkephalin and galanin in the PVN and orexin (ORX) and MCH in the PFLH. Moreover, HFD is associated with an increase in the number of CXCR4^+^ cells in the PVN. Conversely, in the arcuate nucleus, the levels of CXCL12 and CXCR4 were too low to be detected. The ICV injection of CXCL12, next to the hypothalamus, is able to recapitulate the effects of HFD consumption: (1) it reduces novelty-induced locomotor activity, as a 5-day HFD feeding period does; (2) it is associated to an increase in gene expression of enkephalin in the PVN; and (3) it induces an acute increase in calorie intake (by overconsumption of the HFD only and no consumption of chow diet, as rats had access to both diets). This last point interestingly suggests that CXCL12 could participate in the control ingestive behavior, especially since some different studies also previously demonstrated that CXCL12 could affect other systems such as the MCH and vasopressin in the hypothalamus or dopamine in the substantia nigra (18, 113, 114). Taken together, these results suggest that CXCL12 could modulate the activity of orexigenic peptide-producing neurons, especially the encephalin neurons in the PVN. This would favor HFD intake and decrease locomotor activity and, thus, participate in HFD-induced weight gain (Figure 3).

The chemokine CCL5, also known as RANTES for Regulated on Activation, Normal T Cell Expressed and Secreted, has already been linked to obesity-associated inflammation in periphery. Indeed, obesity is associated with an increase in CCL5 secretion and gene expression in AT in obese human and mice. There, CCL5 is suspected to mediate the increase in the local accumulation of T cells and macrophages, which is involved in the complex genesis of chronic inflammation (115, 116). In addition, our joint study with Dr. Karine Clément’s team proposed CCL5 as biomarker of weight evolution in patients undergoing bariatric bypass surgery Roux-en-Y as its levels were nearly eightfold higher in the serum of obese patients than in the one of control patients. CCL5 seems to be the only chemokine of which serum levels appear to be correlated with caloric intake in patients undergoing bariatric bypass surgery Roux-en-Y: they rapidly decrease after surgery, as caloric intake decreases and, later on, when patient’s caloric intake and body weight start increasing again, they also increase again (77). This justified further interest in the potential role of CCL5 as a modulator of the activity of hypothalamic neurons regulating food intake and which could possibly promote the establishment and/or development of obesity. Thus, our group characterized its expression profile, both at the peripheral and hypothalamic levels in a model of nutritional obesity in mice. In this model, we found both peripheral and central inflammations, as evidenced by the overexpression of canonical pro-inflammatory mediators in the serum and hypothalamus. CCL5 was also found to be overexpressed in the periphery and in the hypothalamus of obese animals (78). An ICV injection of CCL5 on the expression of orexigenic peptides MCH and ORX seems able to increase the expression of both ORX and MCH, transiently for ORX and in a more stable and persistent manner for MCH. Furthermore, it would appear that CCL5 is capable of depolarizing MCH neurons, thus facilitating their activation (78). These results indeed suggest that CCL5 is acutely able to modify the activity of the hypothalamic orexigenic MCH system. Taken together, these results suggest that the overexpression of CCL5 would promote the overactivation of hypothalamic MCH neurons and thus participate in weight gain (Figure 3). Whether CCL5 is a cause or a consequence of obesity, or if it could effectively disrupt the proper function of the neuropeptidergic circuits of the hypothalamus, which regulate energy balance and promote the establishment of obesity, is nevertheless still matter of investigation.

## Conclusion

As of today, a substantial amount of publications supports that hypothalamic inflammation mediates disruptions in the hypothalamic control of energy homeostasis, especially regarding body weight regulation. If some pathways and cellular actors have been identified, mechanisms are still ill-described and poorly understood.

Chemokines have long been considered as essential mediators of the inflammatory response, but more particularly because of their ability to activate and attract immune cells to the affected site. However, at the central level, the literature has also attributed to chemokines and their receptors, beyond their role in the attraction of leukocytes in the cerebral parenchyma, important roles in neuronal survival as in neurotoxicity, cerebral development, communication between immune and glial cells, and also communication between neurons and glial cells (since all these cell types have been shown to be able to express the actors of the chemokinergic system) and also in neuromodulation (15, 18).

Several studies now support the hypothesis that chemokines are actually able to modulate the activity of certain neurons. We and others were able to demonstrate that their overexpression is able to deregulate the neuropeptidergic systems of the hypothalamus, which participate in the regulation of the energy balance and participate in the development of deregulations of the latter, whether it is an excessive weight loss or gain (Figure 3). Moreover, this does not, in any way, exclude that, under physiological conditions, the chemokines are actors of this regulation. These studies on chemokine central signaling now identify chemokines as novel potential therapeutic targets against deregulations of the energy balance.

## Ethics Statement

The protocols were carried out in accordance with French standard ethical guidelines for laboratory animals and with approval of the Animal Care Committee (Nice-French Riviera, project agreements no. 04042.01 and 04464.01).

## Author Contributions

OLT conceived and wrote the manuscript. OLT also made the figures. OLT and KS realized the bibliography researches. CC, JLN, NB, and CR critically appraised and revised the manuscript and figures. All authors listed made substantial, direct, and intellectual contribution to the work and gave permission for this manuscript to be published.

## Conflict of Interest Statement

The authors declare that the research was conducted in the absence of any commercial or financial relationships that could be construed as a potential conflict of interest.
